# Diagnostic Value of Serum Procollagen III N-Terminal Peptide for Liver Fibrosis in Infantile Cholestasis

**DOI:** 10.3389/fped.2020.00131

**Published:** 2020-03-31

**Authors:** Yingcan Wang, Weihua Pan, Dongying Zhao, Yan Chen, Xuting Chen, Hongping Xia

**Affiliations:** ^1^Department of Neonatology, Xinhua Hospital, Shanghai Jiao Tong University School of Medicine, Shanghai, China; ^2^Department of Pediatric Surgery, Xinhua Hospital, Shanghai Jiao Tong University School of Medicine, Shanghai, China

**Keywords:** cirrhosis, liver fibrosis, infant, procollagen III N-terminal peptide, biliary atresia

## Abstract

**Background:** Several non-invasive markers have been reported as being effective for the assessment of fibrosis in adults with chronic viral hepatitis. The infantile liver is more susceptible to cholestasis, and it is important to promptly evaluate liver fibrosis to guide the clinical treatment. However, the clinical value of these markers in infants with cholestasis remains unknown.

**Aim:** To investigate the correlation between serum laminin (LN), hyaluronic acid (HA), procollagen III N-terminal peptide (PIIINP) level, and liver fibrosis stage in infants with cholestasis.

**Methods:** One hundred and thirty-seven term infants with cholestasis were included. Laparoscopic exploration and cholangiography were performed to diagnose or rule out biliary atresia. Serum LN, HA, and PIIINP were measured prior to laparoscopic exploration. Liver biopsy was performed for all patients. Liver fibrosis was staged on a five-point scale (F0–F4) according to the METAVIR scoring system. The correlation between serum markers and liver fibrosis stage was assessed. A receiver operator characteristic analysis was performed to determine the accuracy of serum markers for predicting the liver fibrosis stage.

**Results:** Serum PIIINP and HA were positively correlated with liver fibrosis stage (*r* = 0.622, *P* < 0.001, and *r* = 0.41, *P* < 0.001, respectively). There was no significant correlation between serum LN and liver fibrosis stage (*P* > 0.05). Serum aspartate aminotransferase, total bilirubin, direct bilirubin, and PIIINP were independently correlated with the fibrosis stage on multivariate ordinal regression analysis. Receiver operating curve (ROC) analysis showed that serum PIIINP was the most effective for the diagnosis of fibrosis grade. The area under the ROC curves (AUROCs) for serum PIIINP for diagnosing fibrosis stages ≥F1, ≥F2, ≥F3, and F4 (cirrhosis) were 0.843, 0.789, 0.82, and 0.891, respectively. The cut-off serum PIIINP value for predicting fibrosis stage ≥F1 was 242.3 ng/mL, with 73.8% sensitivity and 90% specificity. The cut-off value for predicting cirrhosis was 698.7 ng/mL, with 75% sensitivity and 96% specificity.

**Conclusion:** Serum PIIINP is a promising biomarker for predicting liver fibrosis stage, especially cirrhosis. Its assessment is a simple and non-invasive diagnostic method for liver fibrosis in infants with cholestasis.

## Introduction

Cholestasis is a central key manifestation of hepatobiliary disease in patients of all ages. The neonatal liver is more susceptible to cholestasis compared to older children and adults (1). Common causes of infantile cholestasis include biliary atresia, neonatal hepatitis (of which there are many etiologies), choledochal cyst, and other causes. Liver fibrosis is the common pathologic process of all chronic liver diseases, which results from excessive extracellular matrix accumulation (2–6). If effective treatment is not administered promptly, affected children can rapidly develop end-stage liver disease (cirrhosis, liver failure, and eventual death) in the first year of life (7). It is extremely important to evaluate liver fibrosis promptly to guide the clinical diagnosis and treatment and to improve prognosis.

The gold standard for assessment of fibrosis is liver biopsy and it is helpful for identifying the cause of cholestasis and guiding the treatment. However, in some areas, several issues limit its application for monitoring disease course, including risk of injury to the patient, variable accessibility of the damaged section of the liver, sampling errors, and inaccuracy due to inter- and intra-observer variability of pathologic interpretations (8). As a consequence, non-invasive and simple tools to accurately monitor disease course are highly desirable.

In previous studies, several non-invasive markers such as serum laminin (LN), hyaluronic acid (HA), and procollagen III N-terminal peptide (PIIINP) have been reported to be indicators of liver fibrosis in adults with chronic viral hepatitis (9–11). However, the clinical value of these markers in infants with cholestasis remains unknown.

In the present study, we evaluated the clinical significance of serum LN, HA, and PIIINP in diagnosing liver fibrosis in infants with cholestasis.

## Materials and Methods

### Patients

All term infants with cholestasis admitted to Xinhua Hospital Shanghai Jiao Tong University School of Medicine who underwent liver biopsy between July 2016 and July 2019 were enrolled in the present study. Cholestasis was defined as a value of direct bilirubin <1.0 mg/dL if the total bilirubin is <5 mg/dL, or a value of direct bilirubin that represents more than 20% of the total bilirubin if the total bilirubin is <5 mg/dL (12). The inclusion criteria were: (a) age between 1 week and 5 months; (b) diagnosis of cholestasis; and (c) availability of detailed clinical, imaging, and laboratory examination data. The exclusion criteria were: (a) low birth weight (<2,500 g); (b) previous administration of parenteral nutrition; (c) previous acute liver failure, shock, or sepsis; and (d) previous hepatobiliary surgery.

Abdominal ultrasonography, hepatobiliary scintigraphy, and magnetic resonance cholangiopancreatography were routine examinations administered to infants with cholestasis. Laparoscopic exploration and cholangiography were performed to diagnose or rule out biliary atresia when biliary atresia was highly suspected as indicated by imaging examinations, or persistent severe cholestasis was unresponsive to standard medical treatment and biliary atresia could not be ruled out. Biliary atresia was diagnosed when there was obliteration of extrahepatic bile ducts confirmed by cholangiography (13, 14). Kasai surgery was performed in infants with biliary atresia diagnosed by cholangiography, and bile duct irrigation was performed in infants without biliary atresia. Choledochal cyst resection and Roux-en-Y hepaticojejunostomy were performed in infants with choledochal cyst.

### Ethical Considerations

This study was approved by the Ethics Committee of Xinhua Hospital Shanghai Jiao Tong University School of Medicine. The parents of the infants investigated in this study provided written informed consent for their infants' participation.

### Laboratory Detection

Blood samples were obtained from all patients via a routine laboratory check-up prior to laparoscopic exploration. The blood cell counts were determined by automatic blood cell analysis. Serum aspartate aminotransferase (AST), alanine aminotransferase (ALT), gamma-glutamyl transferase (GGT), total bilirubin (TB), and direct bilirubin (DB) were measured using kits supplied by FUJIFILM Wako Pure Chemical Corporation, Osaka, Japan. Serum LN, HA, and PIIINP were quantified using chemiluminescent immunoassay. The kits were supplied by Shenzhen New Industry Biomedical Engineering Company, Shenzhen, China. The procedures were performed according to the manual.

### Liver Histology

All patients underwent laparoscopic exploration and cholangiography, and liver biopsy was performed during laparotomy or laparoscopy. Wedge-shaped tissue was taken from the edge of one lobe of liver. The specimens were routinely formalin-fixed, paraffin-embedded, and stained with hematoxylin-eosin and Masson's trichrome stains. Samples containing at least six portal tracts were defined as adequate. Liver fibrosis was semi-quantitatively evaluated using the METAVIR scoring system (15). Fibrosis (F) was staged on a five-point scale (F0, no fibrosis; F1, portal fibrosis without septa; F2, portal fibrosis and few septa; F3, numerous septa without cirrhosis; F4, cirrhosis). Where there was evidence of more than one stage, the more severe stage was adopted for the analysis. The pathologist was blinded to the results of serum indices of the study subjects.

### Statistical Analyses

Statistical analyses were performed using SPSS version 23.0 for Windows (SPSS Inc., Chicago, IL, USA). Continuous variables were summarized as mean ± standard deviation or median and interquartile range (IQR) (25th and 75th percentile), depending on whether their distributions were normal, whereas categorical variables were summarized as numbers of cases and percentages. Demographic characteristics and laboratory examination results were compared between the fibrosis stages using the Kruskal-Wallis test, chi-square test or Mann–Whitney *U*-test, as appropriate. Correlations between the stage of histological liver fibrosis and serum indices were assessed using a Spearman analysis. Multivariate ordinal regression analysis was performed with fibrosis stage vs. the parameters significantly correlated in the Spearman analysis for identification of independent predictors. Sensitivity of the assays was plotted against false positivity (1-specificity) using receiver operator characteristic (ROC) curves. Tests were considered statistically significant at *P* < 0.05.

## Results

### Characteristics of the Patients

From July 2016 to July 2019, 143 term infants with cholestasis were included, while one infant was excluded because of low birth weight, one because of parenteral nutrition, two because of shock or sepsis, and two because of previous hepatobiliary surgery. Therefore, 137 infants were eligible for statistical analysis, including 74 females (54.0%) and 63 males (46.0%), with a median (IQR) age of 54.0 (42.0–67.0) days. Ninety infants (65.7%) were diagnosed with biliary atresia (four combined with cytomegalovirus infection), 36 (26.3%) with choledochal cyst (one combined with cytomegalovirus infection), four (2.9%) with cytomegalovirus hepatitis. One infant was diagnosed with Alagille syndrome with *de novo* heterozygous mutation (c.532delC) in JAG1. One infant was diagnosed with Zellweger syndrome with two heterozygous mutations in PEX26. One infant was diagnosed with COACH syndrome with two heterozygous mutations in CC2D2A. The other four infants with cholestasis were of indeterminate cause. According to the METAVIR scoring system, there were 30 F0 stage infants, 41 F1 stage infants, 26 F2 stage infants, 28 F3 stage infants, and 12 F4 stage infants.

### Comparisons of Parameters Between the Patients With Different Fibrosis Stages

Table 1 shows the comparison of clinical characteristics and laboratory examination results in patients with different fibrosis stages. There were no statistically significant differences in gender and weight between patients of different fibrosis stages. Age tended to increase with the severity of fibrosis, especially in infants with cirrhosis (*P* < 0.05). The proportion of biliary atresia was higher in infants with higher fibrosis stage (*P* < 0.05). Serum levels of ALT, AST, TB, DB, GGT, PIIINP, and HA were significantly different in infants at different fibrosis stages (*P* < 0.05). There was no significant difference in blood platelet count and serum LN level between any of the groups (*P* > 0.05).

**Table 1:** Comparison of characteristics in patients at different fibrosis stages.

**Characteristics**	**F0**	**F1**	**F2**	**F3**	**F4**	***P*-value**
Number	30	41	26	28	12	
Age (days)^#^	52.0 (35.8, 73.8)	48.0 (37.0, 59.5)	53.5 (32.8, 62.8)	57.0 (46.5, 74.0)	68.0 (55.5, 111.5)	0.008^*^
Males, n (%)	13 (43.3)	17 (41.5)	14 (53.8)	13 (46.4)	6 (50.0)	0.886
Weight (g)^#^	4850 (3650, 5425)	4500 (4250, 5055)	5000 (3938, 5525)	5000 (4350, 5775)	5150 (4575, 6325)	0.295
BA, n (%)	8 (26.7)	25 (61.0)	22 (84.6)	25 (89.3)	10 (83.3)	<0.001^*^
ALT (U/L)^#^	66.0 (31.0, 107.8)	88.0 (24.5, 161.0)	83.5 (48.5, 153.0)	180.5 (88.0, 243.5)	207.5 (154.3, 416.5)	<0.001^*^
AST (U/L)^#^	88.0 (50.8, 176.8)	135.0 (55.5, 206.5)	141.0 (102.8, 249.3)	208.0 (155.8, 355.5)	360.5 (213.8, 620.8)	<0.001^*^
TB (μmol/L)^#^	86.6 (19.5, 147.7)	126.7 (106.0, 160.2)	144.2 (109.3, 167.3)	146.7 (116.7, 167.8)	149.8 (130.5, 170.9)	0.008^*^
DB (μmol/L)^#^	14.2 (5.0, 86.5)	81.8 (24.4, 97.4)	83.7 (71.9, 117.9)	102.9 (85.1, 120.7)	111.6 (84.8, 124.5)	<0.001^*^
GGT (U/L)^#^	268.0 (94.0, 652.8)	207.0 (132.5, 489.0)	463.5 (250.0, 736.3)	405.0 (237.5, 577.8)	523.0 (212.3, 1084.0)	0.042^*^
BPC (×10^9^/L)^#^	368.0 (293.5, 528.8)	352.0 (268.0, 485.0)	403.5 (201.3, 539.5)	387.5 (314.0, 573.8)	350.0 (213.8, 449.3)	0.747
LN (ng/mL)^#^	289.8 (197.1, 384.8)	367.4 (181.9, 525.0)	252.7 (137.8, 426.4)	321.0 (136.6, 576.4)	364.8 (155.4, 537.3)	0.557
HA (ng/mL)^#^	109.7 (78.4, 132.2)	112.0 (88.7, 207.0)	180.2 (135.8, 242.7)	161.3 (122.3, 214.4)	296.8 (206.7, 547.0)	<0.001^*^
PIIINP (ng/mL)^#^	170.9 (110.9, 206.9)	273.7 (178.4, 371.9)	274.9 (210.4, 497.1)	363.2 (324.5, 473.2)	811.9 (483.7, 985.2)	<0.001^*^

# *Results are presented as median and interquartile range (IQR, 25th and 75th percentile), because their distributions are not normal*. *Significant differences were evaluated using the Kruskal–Wallis test or Chi-square test*.

*IQR, interquartile range; BA, biliary atresia; ALT, alanine aminotransferase; AST, aspartate aminotransferase; GGT, gamma-glutamyl transferase; TB, total bilirubin; DB, direct bilirubin; BPC, blood platelet count; LN, laminin; HA, hyaluronic acid; PIIINP, procollagen III N-terminal peptide*.

* *indicates significant difference*.

The Spearman analysis showed a positive correlation between serum ALT, AST, TB, DB, GGT, PIIINP, and HA level and liver fibrosis stage (*r* = 0.409, 0.446, 0.299, 0.471, 0.247, 0.622, and 0.41, respectively, *P* < 0.005), but there was no significant correlation between serum LN level and fibrosis stage (*P* > 0.05). The Mann–Whitney *U*-test showed that serum HA level increased more in F2/F3/F4 than in F0/F1 (189.6 [139.0–282.1] vs. 111.5 [83.1–185.7] ng/mL, *Z* = −4.64, *P* < 0.001).

**Figure 1:**
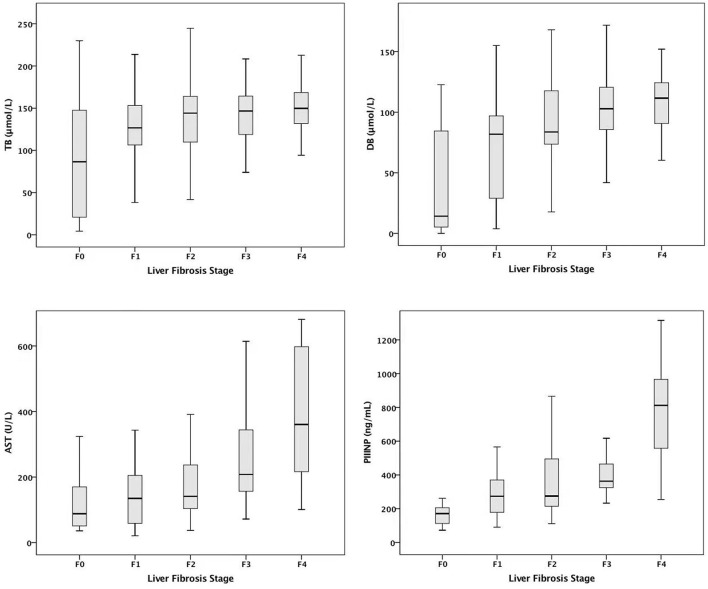
Serum TB, DB, AST, and PIIINP levels in infants at different fibrosis stages. AST, aspartate aminotransferase; TB, total bilirubin; DB, direct bilirubin; PIIINP, procollagen III N-terminal peptide.

Multivariate ordinal regression analysis showed that serum AST, TB, DB, and PIIINP were independently correlated with fibrosis stage adjusted for age and biliary atresia, as shown in Table 2. Box-plot representation of serum TB, DB, AST, and PIIINP levels in infants at different fibrosis stages are shown in Figure 1.

**Table 2:** Multivariate ordinal regression analysis for serum AST, TB, DB, and PIIINP with fibrosis stage after adjusting for age and biliary atresia.

**Parameter**	**Adjusted OR (95% CI)**	***P*-value**
ALT	1.0025 (0.9996–1.0055)	0.09
AST	1.0024 (1.0004–1.0045)	0.021^*^
TB	1.0083 (1.0023–1.0143)	0.006^*^
DB	1.0153 (1.0062–1.0245)	0.001^*^
GGT	1.0003 (0.9997–1.001)	0.316
HA	1.0001 (0.9997–1.0005)	0.653
PIIINP	1.0054 (1.0035–1.0073)	<0.001^*^

*OR, odds ratio; CI, confidence interval; ALT, alanine aminotransferase; AST, aspartate aminotransferase; GGT, gamma-glutamyl transferase; TB, total bilirubin; DB, direct bilirubin; LN, laminin; HA, hyaluronic acid; PIIINP, procollagen III N-terminal peptide*.

* *indicates significant difference*.

### Diagnostic Performance of Indices Comparison

**Figure 2:**
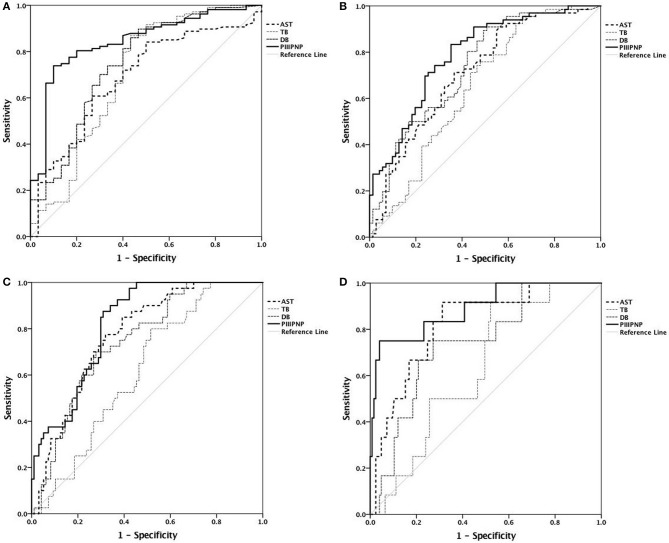
Receiver operating characteristic curves. **(A)** Diagnosis of stage ≥F1; **(B)** diagnosis of stage ≥F2; **(C)** diagnosis of stage ≥F3; **(D)** diagnosis of stage F4.

ROC analysis revealed that serum PIIINP was the best diagnostic indicator of the stage of fibrosis, with an area under the curve of 0.843, 0.789, 0.82, and 0.891 for diagnosis of stages ≥F1, ≥F2, ≥F3, and F4, respectively. More details are shown in Table 3 and Figure 2. The cut-off values were set as the levels that resulted in the maximum sum of sensitivity and specificity. The cut-off serum PIIINP value for predicting stages ≥F1 was 242.3 ng/mL with 73.8% sensitivity and 90% specificity, whereas the cut-off value for predicting F4 was 698.7 ng/mL with 75% sensitivity and 96% specificity.

**Table 3:** Diagnostic performance of serum AST, TB, DB, and PIIINP for staging fibrosis grades.

**Indices**	**AUROC (95% CI)**			
	**≥F1**	**≥F2**	**≥F3**	**F4**
AST	0.687 (0.582–0.793)	0.713 (0.627–0.799)	0.773 (0.694–0.852)	0.822 (0.711–0.934)
TB	0.7 (0.576–0.824)	0.648 (0.555–0.74)	0.617 (0.522–0.711)	0.635 (0.506–0.765)
DB	0.746 (0.638–0.854)	0.737 (0.655–0.82)	0.746 (0.662–0.83)	0.739 (0.605–0.872)
PIIINP	0.843 (0.766–0.92)	0.789 (0.714–0.864)	0.82 (0.752–0.889)	0.891 (0.788–0.995)

*AUROC, area under receiver operating curve; CI, confidence interval; AST, aspartate aminotransferase; TB, total bilirubin; DB, direct bilirubin; PIIINP, procollagen III N-terminal peptide*.

## Discussion

In the present study, we found that serum PIIINP was the best diagnostic indicator of the stage of fibrosis in infants with cholestasis. As far as we know, this is the first study to evaluate serum PIIINP for liver fibrosis in infants.

In newborns and infants, congenital biliary atresia, choledochal cyst, and long-term cholestasis cause liver fibrosis (even cirrhosis) and may result in serious liver dysfunction. The severity of liver fibrosis is known to be a good indicator for determining the prognosis and optimal treatment of cholestasis (16, 17). Laparoscopic exploration should be performed as early as possible in infants with increasing fibrosis, if biliary atresia cannot be ruled out. In addition, liver fibrosis evaluation after surgery is helpful to evaluate the condition of the liver, and can guide the following treatment (18, 19).

Advanced chronic liver disease has been known to be accompanied by thrombocytopenia (20). A negative correlation has been reported to exist between the stage of liver fibrosis and blood platelet count, especially in patients with hepatitis C and non-alcoholic fatty liver disease (21). The platelet count has been used in scoring systems used for various liver diseases (22). However, in this study, there was no relationship between blood platelet count and liver fibrosis. This may because the infants in this study seldom had portal hypertension and enlargement of the spleen due to the short duration of cholestasis.

Cholestasis of different etiologies can cause different degrees of liver injury, manifested as abnormal liver function. Generally, liver function is more impaired with progression of the disease. ALT, AST, bilirubin, and GGT are well-known predictors of liver disease progression, reflecting the severity of hepatocellular injury and cholestasis. Our results showed that serum ALT, AST, TB, DB, and GGT were all significantly positively correlated with liver fibrosis stage. However, multivariate analysis showed that serum ALT and GGT were not independently associated with fibrosis stage when adjusted for age and biliary atresia. Mansoor et al. reported that the AST/ALT ratio and the AST to platelet ratio index (APRI), which are used to identify advanced fibrosis in adult patients, may not be accurate for predicting advanced fibrosis in children (23, 24). In our study, AST showed good diagnostic performance for cirrhosis (F4) with an AUROC of 0.822. This may be because the increased serum AST may reflect hepatocyte inflammation, damage, or cholestasis that is related to the occurrence and progression of liver fibrosis.

LN is one of the main glycoproteins of the basement membrane and it is synthesized by hepatocytes and sinusoidal cells. Serum LN levels have shown a close positive and linear correlation with liver fibrosis stage in adults with chronic hepatitis (25, 26). In our study, there was no significant difference between infants at different fibrosis stages, and there was no significant correlation between serum LN level and fibrosis stage. The deposition of LN may result in sinusoid capillarization, which has a major correlation with portal hypertension (27). Kong et al. conducted a study on 39 adults with cirrhosis of various causes, and the results showed that serum LN levels decreased markedly immediately after splenectomy compared to pre-surgery, suggesting that LN may be mainly related to portal hypertension rather than liver fibrosis (28).

HA is a glycosaminoglycan synthesized by hepatic stellate cells (HSCs) and it is a component of the extra-cellular matrix (ECM). Li et al. reported that serum HA showed positive correlation with liver fibrosis stage in adults with chronic hepatitis B (26). Matsue et al. found that HA was effective in differentiating between F0/F1 and F2/F3/F4 in chronic hepatitis C patients (29). Similarly, in our study, serum HA levels increased more in F2/F3/F4 patients compared to F0/F1 patients. However, multivariate analysis showed that serum HA level was not independently associated with the fibrosis stage. In the study by Valva et al. HA was only related to worse fibrosis stages in adults with chronic hepatitis C, but not in children (30).

PIIINP is a cleavage product of collagen precursor and has been the most closely studied non-invasive marker of liver fibrosis in chronic hepatitis C and primary biliary cirrhosis (10, 31). Many studies have showed a significant correlation between serum PIIINP level and histological liver fibrosis stage in adults (32–36). Leroy et al. (37) reported that serum PIIINP level was a good test of liver fibrosis stage in patients with chronic hepatitis C, with an AUROC value of 0.88 for diagnosis of stages ≥F3. Our results showed that serum PIIINP level increased significantly with the severity of liver fibrosis, and the correlation between serum PIIINP level and liver fibrosis stage was strong, positive, and linear. Serum PIIINP was an independent predictor of liver fibrosis stage by multivariate ordinal regression analysis. These findings suggest that infants with higher serum PIIINP levels might have a higher liver fibrosis stage, which is compatible with other reports in the literature regarding adults.

ROC analysis showed that serum PIIINP was of a moderate diagnostic value for fibrosis stage, and better than serum AST, TB, and DB. Serum PIIINP was most accurate for diagnosing cirrhosis, with the largest AUROC of 0.891. While the diagnosis of ≥F1 came second with an AUROC of 0.843, the accuracy was poorest for diagnosis of stages ≥F2 with an AUROC of 0.789. These results indicated that serum PIIINP was more useful for detecting liver fibrosis and cirrhosis than discriminating between mild and moderate fibrosis. We identified serum PIIINP cutoff values as 242.3 ng/mL for predicting stages ≥F1 and 698.7 ng/mL for predicting cirrhosis.

### Limitations

Our sample size was relatively limited and infants with different diseases were included in the study. Thus, we do not believe that serum PIIINP can replace liver biopsy for the assessment of liver fibrosis at this point in time. Further prospective studies are required to determine the potential utility of serum PIIINP testing in guiding treatment decisions and monitoring disease course.

## Conclusion

Serum PIIINP level showed moderate diagnostic value as an independent predictor of liver fibrosis stage. Its assessment is a simple and non-invasive diagnostic method and it is a promising biomarker for predicting liver fibrosis stage, especially cirrhosis in infants with cholestasis.

## Data Availability Statement

The datasets generated for this study are available on request to the corresponding author.

## Ethics Statement

The studies involving human participants were reviewed and approved by Ethics Committee of Xin Hua Hospital Affiliated to Shanghai Jiao Tong University School of Medicine. Written informed consent to participate in this study was provided by the participants' legal guardian/next of kin.

## Author Contributions

YW contributed to the acquisition, analysis, and interpretation of data for the work. WP performed the operations and approved the version to be submitted. DZ and YC contributed to analysis and interpretation of the data. XC contributed to liver fibrosis evaluation. HX contributed to the design of the work and revised the paper critically for important intellectual content. All authors have read and approved the final version to be published.

### Conflict of Interest

The authors declare that the research was conducted in the absence of any commercial or financial relationships that could be construed as a potential conflict of interest.
